# Effectiveness of the Healthy Lifestyle Promotion Program for Yaquis with Obesity and Risk of Diabetes in the Short and Medium Term: A Translational Study

**DOI:** 10.1155/2020/6320402

**Published:** 2020-09-29

**Authors:** Alejandro Arturo Castro-Juarez, Araceli Serna-Gutiérrez, Norma Alicia Dórame-López, Mariela Solano-Morales, Ana Cristina Gallegos-Aguilar, Rolando Giovanni Díaz-Zavala, Heliodoro Alemán-Mateo, Rene Urquidez-Romero, Fernanda Campa-Quijada, Diana Marcela Valenzuela-Guzmán, Julián Esparza-Romero

**Affiliations:** ^1^Diabetes Research Unit, Department of Public Nutrition and Health, Nutrition Coordination, Research Center for Food and Development (CIAD), A.C., Hermosillo, Sonora 83304, Mexico; ^2^Sociocultural Department, Technological Institute of Sonora, Cd. Obregon, Sonora 85137, Mexico; ^3^Nutrition Health Promotion Center, Department of Chemical and Biological Sciences, University of Sonora, Hermosillo, Sonora 83000, Mexico; ^4^Department of Nutrition and Metabolism, Nutrition Coordination, Research Center for Food and Development (CIAD), A.C., Hermosillo, Sonora 83304, Mexico; ^5^Department of Health Sciences, Nutrition Program, Autonomous University of Ciudad Juarez, Cd. Juarez, Chihuahua 32315, Mexico

## Abstract

Type 2 diabetes (T2D) is a public health problem worldwide, and the main risk factor for its development is obesity. The Yaqui ethnic group of Sonora has serious obesity problems, resulting in an increased risk of T2D in its inhabitants. The objective of this study was to evaluate the effectiveness of a health promotion program on obesity parameters and cardiovascular risk factors in short- (6 months) and medium-term periods (12 months) in indigenous Yaquis of Sonora. The design is a translational clinical study of a single cohort with prepost intervention measurements in a sample of 93 subjects. The effectiveness of the program was evaluated by comparing obesity parameters, metabolic markers, and physical activity 6 and 12 months with those measured under basal conditions using a paired *t*-test or Wilcoxon rank-sum test. The short-term retention percentage was 58.0%. There was a decrease in body weight (Δ = −3.9 kg, *p* ≤ 0.05) and other obesity parameters, and an increase in physical activity and improvements in metabolic markers (*p* ≤ 0.05) was observed. Similar findings were obtained for the medium-term period; body weight loss was also -3.9 kg (*p* ≤ 0.05). The short and medium-term results of the program showed improvements in the obesity parameters and other cardiovascular risk factors of the participants. These results support the effectiveness of the program and its translation in this ethnic group.

## 1. Introduction

Obesity is a worldwide epidemic and is the main risk factor for the development of type 2 diabetes (T2D) [1, 2]. The prevalence of obesity has increased considerably in recent decades; currently, the prevalence of overweight in adults is 39%, and that of obesity is 13%, representing 1900 million people with these conditions worldwide [2]. In the same vein, T2D is considered a public health crisis that affects all social and ethnic groups worldwide [3]. It is currently estimated that T2D affects 463 million people, causing 4.2 million deaths per year. In addition, it is estimated that by 2045 there will be 700 million people with this condition [4]. T2D leads to an increased risk of cardiovascular disease, vision loss, kidney disease, amputations, disability, and mortality [5]. In 2019, it was estimated that Mexico ranked sixth in terms of the prevalence of T2D, with 12.8 million of its inhabitants with this disease and an expected 22.3 million in 2045 [4]. The high prevalence of chronic diseases and their comorbidities are also present in indigenous groups, and due to their vulnerability, this problem can be worse in indigenous than in nonindigenous populations.

Indigenous populations around the world have well-marked disadvantages and are characterized by poverty and marginalization, which directly affect their health status. Some of these ethnic groups have the highest rates of communicable and noncommunicable diseases, so their life expectancy is five years shorter than that of nonindigenous populations [6–8]. Decades ago, the problem of T2D practically did not exist, or its prevalence was very low in some of the indigenous Mexican communities [9, 10]. However, there has been an explosive increase in the prevalence and incidence of T2D in particular ethnic groups, due mainly to acculturation and westernization processes, modifying their lifestyle. There has been an increase in the caloric intake of fats and refined sugars and a decrease in vigorous physical activity in these populations [9–11]. This change in traditional lifestyle explains why some indigenous groups across the country and other Latin American indigenous groups have a high prevalence of obesity and T2D [10–15].

One of the most representative ethnic groups in northern Mexico is the Yaqui tribe, which resides in the state of Sonora and is distributed among eight traditional villages (Vícam, Pótam, Loma de Bácum, Loma de Guamúchil, Tórim, Rahum, Huiribis, and Belem) [16]. A study conducted in the Yaqui community reported high prevalences of overweight and obesity of 25.2% and 42.9%, respectively, in this ethnic group and other related diseases, such as hypertension, which implies the development of chronic diseases among its inhabitants [16]. In 2008, a prevalence of T2D of 18.3% was reported in indigenous Yaqui individuals. In that study, it was reported that the diet of this indigenous group predisposes individuals to overweight and obesity and other comorbidities due to the high intake of total and saturated fats [17]. Another study showed how the activities carried out by this tribe have changed; agriculture changed from using a traditional manual method to using a mechanized method, in addition to sedentary activities being adopted within the community, which may have caused a decrease in the intensity of physical activity performed [18]. This indigenous group has serious obesity problems that increase the risk of developing T2D, so it is necessary to carry out intervention strategies through health prevention and promotion programs in this ethnic group. We believe that early intervention in obese subjects with a high risk of diabetes will substantially modify the risk of developing T2D and possibly other chronic diseases.

The prevention of T2D should be a priority in public health [1]. Randomized controlled clinical studies have reported solid and consistent evidence that T2D and its related risk factors can be prevented or delayed through lifestyle modification in subjects with obesity [19–22]. These studies have reported a reduction of up to 58% in the incidence of T2D through weight loss, physical activity, and healthy eating [22]. The challenge is to translate the evidence of these efficacy trials to effectiveness in an open community through research studies with translational applications. The National Diabetes Prevention Program (NDPP) has been structured based on the evidence shown by the Diabetes Prevention Program (DPP) [23]. This program has been shown through translation studies in organizations, health institutions, worker groups, schools, churches, and other communities to prevent the development of T2D in subjects with obesity and diabetes risk [23]. Some studies in which the DPP protocol was implemented have found improvements in obesity parameters and metabolic risk markers, which contribute to the prevention or delay of the development of T2D. In addition, some studies have found short- and medium-term improvements in the parameters evaluated [24–26]. However, information on the effectiveness of programs in the indigenous population is limited.

For this reason, the main objective was to evaluate the effectiveness of a healthy lifestyle promotion program for Yaquis based on the NDPP on obesity parameters and cardiovascular risk factors in the short and medium term in Yaquis adults from Sonora, Mexico.

## 2. Materials and Methods

### 2.1. Study Design

The study design is clinical with a translational application in a single cohort with pre-post intervention measurements. Adults with overweight/obesity and diabetes risk from the Yaqui tribe of the state of Sonora from two villages, Loma de Guamúchil and Tórim, located in the municipalities of Cajeme and Guaymas, respectively, were included. The study began in July 2018 with the recruitment of participants. The implementation of the program lasted from August 2018 to September 2019. The study protocol was approved by the Ethics Committee of the Research Center for Food and Development CIAD, A.C., with approval number CE/008-1/2018. Participants signed the informed letter consent.

### 2.2. Sample Size

The sample size was calculated based on the change in body weight (Δ) from the beginning to the end of the program. The sample size was obtained with the paired *t*-test formula using data from a pilot study. For this, we considered a mean body weight loss of 5.2 kg and a standard deviation of 3.2, with a significance level of 0.05 and a power of 0.8. With these data, we found that a sample size of three subjects was sufficient. Sample size calculations were also analyzed with fasting glucose and triglyceride levels from the pilot study with the purpose of ensuring a sufficient sample size for these variables. The mean for fasting glucose loss was 27.5 mg/dL with a standard deviation of 7.8, and for triglycerides, the mean loss was 14.4 mg/dL with a standard deviation of 36.7. Similarly, the level of significance used was 0.05, and the power was 0.8. With these calculations, the sample size required was 51 subjects. However, as our study is translational research, we decided to increase the sample size to guarantee the results of the comparisons of the outcomes explored. Therefore, any sample size ≥ 51 subjects was feasible.

### 2.3. Recruitment and Eligibility Criteria

Recruitment was prior to the implementation of the program. It consisted of visits to the homes of potential participants from the villages of Loma de Guamúchil and Tórim, as well as announcements that were published in the dispensaries of the communities. The study protocol, objectives, and benefits of the intervention program were explained. People who showed interest in participating were screened using anthropometry and clinical sociodemographic history questionnaires [27], and the FINDRISC (Finnish Diabetes Risk Score) questionnaire was used to identify subjects at a potential risk of T2D [28]. Subjects who met the following inclusion criteria were included: men and women between 20 and 65 years of age, overweight/obese (BMI ≥ 25 kg/m^2^), at risk of T2D according to the FINDRISC questionnaire (score ≥ 12), being of Yaqui ethnicity, wanting to participate, and signing the informed consent form. Subjects with a previous diagnosis of diabetes, uncontrolled hypertension (≥160/100), and other serious illnesses according to self-report and recorded in the clinical history, pregnant and/or breastfeeding women, subjects with physical activity limitations, subjects who participated in a similar program, and subjects undergoing pharmacological treatment for obesity, for glucose tolerance, and for lipid profile were excluded.

### 2.4. Intervention Protocol

#### 2.4.1. Adaptation Program

The program implemented was the NDPP [29, 30], and the protocol of this program was adapted according to the characteristics of the Yaqui ethnic group obtained through previous studies. The foods with the highest consumption and the main activities carried out by this indigenous group were known [18, 31]. Considering their particular pattern of lifestyle, some formats were modified (manuals of the participants and the coach of program), as was the content of the program, to improve the understanding of the participants. In this way, the program was adapted so that it would be culturally accepted by the community, and thus, the “Healthy Lifestyle Promotion Program for Yaquis with Obesity and Diabetes Risk (PREVISY, by its acronym in Spanish which means Programa de Estilo de Vida Saludable para Yaquis)” was formally established.

#### 2.4.2. Intervention Program

The intervention program consisted of two phases. The first was an intensive phase (6 months). The lifestyle coaches who participated had academic training in the area of health (bachelor's and master's in nutrition). Lifestyle coaches were updated on obesity and diabetes issues and were trained for the intervention program. During the intensive phase, 16 group sessions (see Table 1) (with 10-15 people groups) were given weekly, with their respective manuals [32]. These were informative sessions to encourage the adoption of a healthy lifestyle through consuming healthy food, reducing the consumption of fats in food, avoiding foods with a high content of calories, and increasing physical activity and behavioral changes. The objective of this phase was a loss of body weight ≥ 7% and to perform at least 150 minutes of physical activity per week (moderate-to-vigorous). The second phase was the maintenance phase, which had a duration of 6 months. Here, the participation of lifestyle coaches was also important to increase adherence to the program and avoid dropout. The sessions implemented in this phase were held monthly; that is, there were specifically 6 sessions with their respective manuals [33] (see Table 1). The objective at this stage was to transition to the healthy habits learned in the first phase and to maintain them in the medium term. PREVISY sessions were held in community centers and clinics. Sessions were between 45 and 60 minutes using Microsoft® PowerPoint® slides. Before each session, the body weight of each participant was measured, and the physical activity they performed was recorded. The manuals for each session were delivered, and the development of the topic corresponding to each session began. There was also an additional session on holidays and personalized nutritional guidance for participants who wanted it. Participants who attended ≥80% of the sessions (≥13 sessions in the intensive phase and ≥5 sessions in the maintenance phase) were considered program completers [34].

#### 2.4.3. Strategies to Improve Adherence

The PREVISY was implemented with flexible schedules, the sessions were held on weekends (Friday, Saturday and Sunday), and a schedule was established with the dates of the sessions so that there will be no problems with holidays. The participant's manual [32, 33] was simplified to improve understanding of the topics taught, and additional support was provided to people with difficulties in reading and writing in Spanish. Participants received a reminder by phone, text message, and home visit to attend the sessions. If participants missed a session, they had the opportunity to make it up in the next session. Finally, as an additional motivation, monthly raffles for healthy food baskets were performed for the attending subjects.

### 2.5. Measurement of the Variables of Interest

To see the effectiveness of the PREVISY, the measurements were made at the beginning of the study (basal: before starting the intervention program) and at 6 and 12 months. Comparisons were made of all parameters evaluated by the changes between basal and 6 months and between basal and 12 months. These measurements were made in the medical units of the two villages. The subjects received indications for the measurements and arrived in the morning after a 12-hour fast for the respective evaluations. In the case of biochemical parameters, a peripheral venous blood sample of 8 ml was obtained following the methodology reported for its extraction [35]. The samples were transported and analyzed in the laboratory of the Diabetes Research Unit of the Department of Public Nutrition and Health, located at the Research Center for Food and Development CIAD, A.C.

### 2.6. Primary Outcome

Body weight (kg) was obtained using a digital scale with a capacity of 200 kg ± 100 g (Seca 813), according to the established methodology [16, 27].

### 2.7. Secondary Outcomes

#### 2.7.1. Anthropometry, Physical Measurements, and Body Composition

Body mass index (BMI) was calculated by dividing weight in kilograms by the square of height in meters [16, 27]. The height (m) was measured using a portable stadiometer (Seca 213) with an error range of 0.05 mm [16, 27]. Waist circumference (cm) was measured with the subject standing, using the midpoint reference by means of a graduated flexible anthropometric tape in mm (GÜLICK, with a scale from 0 to 150 cm), based on the technique proposed by the International Society for the Advancement of Kinanthropometry (ISAK) [16]. The diagnoses of overweight, obesity, and central obesity were performed according to the World Health Organization WHO criteria [36]. The measurement of the percentage of body fat (%) was carried out by a tetrapolar impedance plethysmograph Quantum II, model BIA-103. All BIA measurements were performed according to the protocol reported by Lukaski et al. and Rising et al. [37, 38]. Systolic and diastolic blood pressure was determined with a digital automatic device (Omron, model HEM-907XL) according to the established technique [39, 40]. With respect to physical activity (minutes/week) [41], baseline physical activity was obtained with the screening questionnaires; here, each participant was asked the number of minutes per week that he/she carried out moderate-to-vigorous intensity physical activity. The physical activity carried out at 6 and 12 months was obtained with the physical activity record that the participants self-recorded during the program.

#### 2.7.2. Biochemical Parameters

Fasting glucose levels (mg/dL) were obtained using the peripheral venous blood sample and the Randox® GOD-PAP technique, total cholesterol (mg/dL) was determined by the enzymatic endpoint method of the Randox® kit, HDL cholesterol (mg/dL) was determined using the Randox® HDL-c precipitation technique, LDL cholesterol (mg/dL) was calculated from the formula of Friedewald et al. [42], and triglyceride levels (mg/dL) were determined using the enzymatic end metric colorimetric method of Randox® GPO-PAP Triglycerides [35].

### 2.8. Statistical Analysis

The characteristics and data of the participants are presented as the means and standard deviation (means ± SD) for continuous variables with a normal distribution and as the median and interquartile range [median (p25, p75)] for continuous variables with a nonnormal distribution. Categorical variables are presented percentages (%). Paired *t*-tests or Wilcoxon rank-sum tests were performed to make comparisons of changes in the primary and secondary outcomes between baseline and 6 and 12 months (*p* ≤ 0.05). These comparisons were made by completers or a protocol analysis (PA) and an intention-to-treat analysis (ITT). An effort was made to obtain the measures of all participants (completers and noncompleters) at 6 and 12 months for the ITT. Some noncompleter subjects did not participate in the 6- and 12-month evaluations, so baseline or the last reported values were taken for these subjects (4 subjects in the intensive phase and 8 subjects in the maintenance phase). If the subjects could not continue in the program due to violation of an inclusion criterion during the PREVISY, they were excluded from the analysis (one subject). The analyses were carried out with the Stata Statistical Software (version 12., StataCorp LP, College Station, TX, USA).

## 3. Results

**Figure 1:**
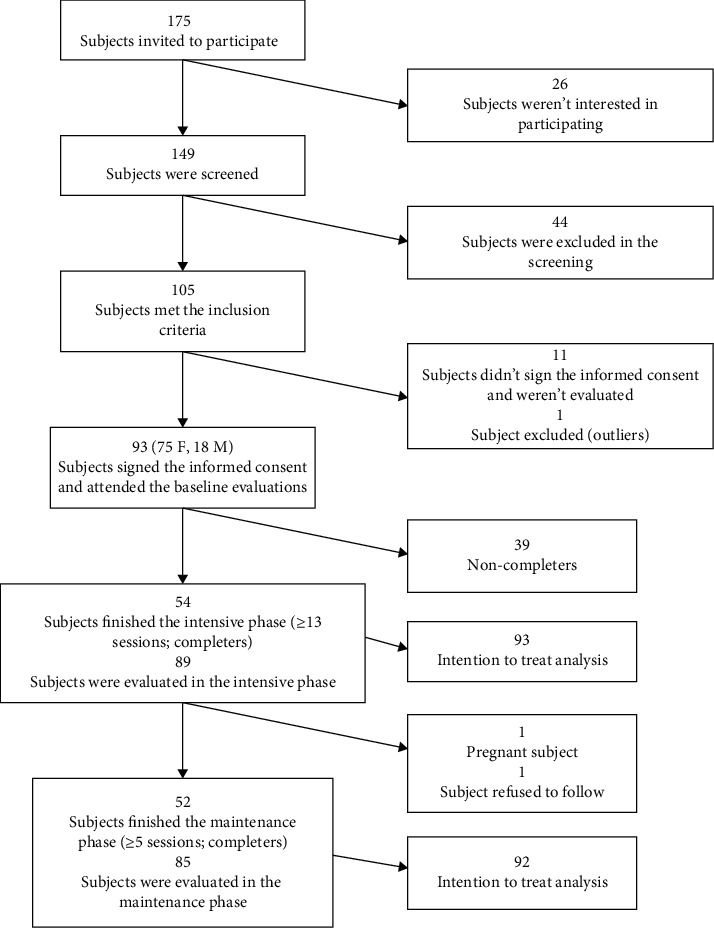
Recruitment and participation of subjects in the intensive (6 months) and maintenance phase (12 months).

Figure 1 summarizes the trajectory of subject participation in the PREVISY. The number of subjects invited, selected, and excluded is shown (see Figure 1). In total, 93 subjects were included from the beginning (75 women and 18 men). The intensive phase was completed by 54 subjects, representing a 58.0% retention. Some major reasons for not having full participation were lack of time, family problems, and work. Fifty-two subjects completed the maintenance phase, representing a 55.9% retention, as shown in Figure 1. One of the participants became pregnant, and another decided not to continue in the maintenance phase. Regarding the percentage of subjects evaluated at the end of the intensive and maintenance phases, this number was considered high, since 95.7% and 91.4% of the subjects were evaluated in the respective phases, regardless of whether they had completed both phases of the program.

The sociodemographic and clinical characteristics of the participants are shown in Table 2. Comparisons of the subjects who completed and did not complete the PREVISY are shown (see Table 2). In this program, there was a predominant percentage of female participants (80.6%). The average age of the participants was 39.5 ± 11.2 years (young adult subjects). Most of the Yaqui community speak and write the Spanish language (see Table 2). Almost three quarters of them were considered to have a basic education, and the majority were married or in free unions. Some characteristics that represent a risk for the development of T2D were also observed. According to the inclusion criteria, all participants were overweight or obese and had a high risk of diabetes, according to the FINDRISC questionnaire, but it was also observed that 98.9% suffered from central obesity, most had a family history of diabetes (91.4%), and 14.0% of the participants suffered from hypertension (previous diagnosis), a risk factor for the development of T2D.

With respect to the comparison analysis between completers and noncompleters, differences were found in the marital status of the participants, where a greater number of people were married or in free union in the group of noncompleter subjects than in the group of completer subjects (*p* ≤ 0.05). In general, Table 2 shows homogeneity when comparing completer and noncompleter subjects, thus emphasizing the success of the program. The recruitment of our study was very strict about identifying subjects who were at risk of T2D. The information presented in Table 2 shows the risk values for the obesity parameters (waist circumference, BMI, and high percentage of body fat). Average fasting glucose was above normal levels at 109.6 ± 26.2 mg/dL. The median amount of physical activity at moderate-to-vigorous intensity was 0 (0, 0) minutes/week, which reflects the degree of the sedentary lifestyle of the participants. For a better understanding, the average minutes/week of moderate-to-vigorous physical activity was calculated; it was 6.0 ± 23.1 minutes/week for all participants and 8.3 ± 27.5 and 2.8 ± 14.6 minutes/week for completer and non-completer subjects, respectively.

**Figure 2:**
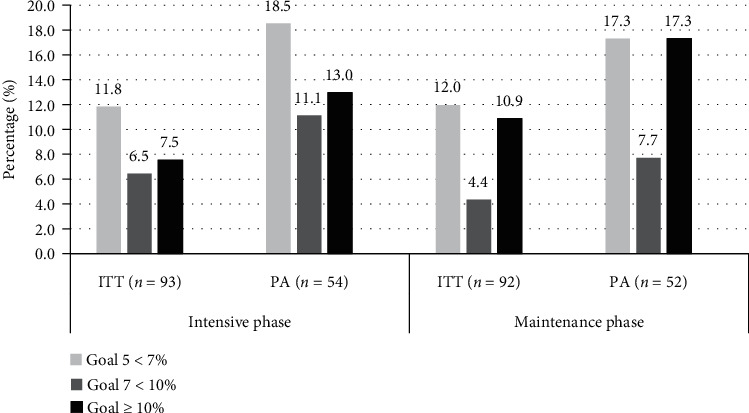
Goal of the loss of body weight achieved by the participants in the intensive and maintenance phase of the program. ITT: intention to treat analysis; PA: protocol analysis (completers).

The PREVISY has two main objectives: one is to reduce body weight, by establishing a loss percentage goal, and the other is to increase physical activity, where participants must perform at least 150 minutes/week of moderate-to-vigorous intensity.  Figure 2 shows the different objectives of body weight loss (5 < 7%, 7 < 10% and ≥10%) achieved by the participants by ITT and PA in both phases of the PREVISY. According to the ITT, at the end of the intensive phase, 25.8% of the participants achieved the body weight goal of ≥5%. At the end of the maintenance phase, 27.4% of the participants achieved the body weight loss goal of ≥5%. On the other hand, according to the PA, 42.6% of completers had a reduction in body weight ≥ 5% of their initial weight at the end of the first phase. In the maintenance phase, 42.3% of the completers lost ≥5% of their baseline body weight.

**Figure 3:**
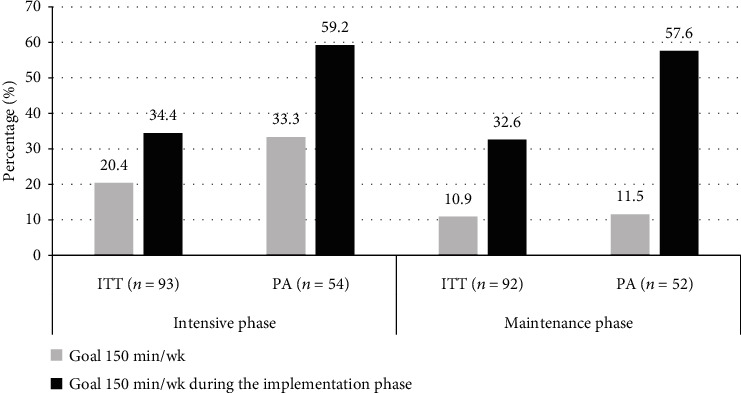
Goal of physical activity achieved by the participants in the intensive and maintenance phase of the program. ITT: intention to treat analysis; PA: protocol analysis (completers).

Figure 3 shows the participants who achieved the physical activity goal at the end of both phases, as well as those who were successful at some point in each phase. According to the ITT, in the intensive phase, 20.4% of the subjects achieved the goal, and 34.4% achieved it at some point during this phase. For the maintenance phase, the numbers decreased by 10.9% and 32.6%, respectively. On the other hand, according to the PA, in the intensive phase, 33.3% of the completers group achieved the objective, and 59.2% achieved it at some point in this phase. For the maintenance phase, 11.5% of completers maintained the goal of 150 minutes/week and 57.6% of completers maintained the goal at some point during this phase.

Tables 3 and 4 show the changes in obesity parameters, metabolic markers, and physical activity according to the ITT and PA, respectively. The differences (Δ) are shown with respect to the baseline measurements; Table 3 shows a comparison of baseline measurements vs. measurements at the end of the intensive phase and another comparison of baseline measurements vs. measurements at the end of the maintenance phase. The ITT showed a loss in body weight of 2.0 ± 4.3 kg (*p* ≤ 0.05) in the intensive phase, representing a loss of 2.3% of the initial body weight. This significant loss of body weight remained in the maintenance phase (1.9 ± 5.1 kg; 2.2%) (*p* ≤ 0.05). With respect to the change in waist circumference and BMI, significant reductions in the intensive and maintenance phases were observed with respect to the baseline measurements (*p* ≤ 0.05). There were also favorable results according to the ITT in metabolic markers, since it significantly improved total cholesterol (-8.5 mg/dL), LDL-c (-9.9 mg/dL), HDL-c (+4.2 mg/dL), triglycerides (-14.8 mg/dL), and fasting glucose (-7.7 mg/dL) at the end of the intensive phase. However, at the end of the maintenance phase, only significant reductions were found in fasting glucose levels (-8.9 mg/dL, *p* ≤ 0.05). For physical activity according to the ITT, there was a significant increase in the minutes/week of the participants (*p* ≤ 0.05); however, it is not seen directly in the table; this increase was 68.4 ± 131.9 minutes/week at the end of the first phase and 29.0 ± 74.5 minutes/week at the end of maintenance phase.

By PA, there were more noticeable improvements than those obtained by ITT. For example, the body weight loss was 3.9 ± 4.5 kg at the end of the intensive phase (*p* ≤ 0.05), representing a loss of body weight of 4.5%. As can be seen, the mean body weight loss was the same at the end of the maintenance phase with 3.9 ± 6.2 kg (*p* ≤ 0.05), resulting in a loss of body weight also of 4.5%. In the intensive phase, there were improvements in waist circumference (-4.2 cm), BMI (-1.5 kg/m^2^), and metabolic markers such as total cholesterol (-12.6 mg/dL), LDL-c (-13.6 mg/dL), triglycerides (-22.7 mg/dL), fasting glucose (-10.7 mg/dL), and HDL-c (+6.0 mg/dL) (*p* ≤ 0.05). These improvements were maintained at the end of the maintenance phase with improvements in waist circumference (-3.6 cm), BMI (-1.5 kg/m^2^), and fasting glucose (-14.4 mg/dL) (*p* ≤ 0.05). However, improvements in the lipid profile were not significant in the medium term (*p* > 0.05). In terms of physical activity, in the two phases, the mean increase was 106.3 ± 155.9 minutes/week in the first phase. However, the participants did not retain the number of minutes achieved at the end of the maintenance phase; the mean increase was 30.3 ± 66.7 minutes/week (*p* ≤ 0.05) (see Table 4).

Comparison of body fat percentages according to the goal of body weight loss achieved by the participants during the PREVISY is provided in supplementary material (see table S1). We carried out a subanalysis to compare the parameters of obesity, metabolic markers, and physical activity, categorizing the completer subjects by overweight and obesity. We also compared the parameters mentioned for diabetes risk (moderate risk and high and very high risk). In both subanalyses, it can be seen that there is a greater benefit in subjects classified as obese and with a higher risk of diabetes (see tables S2-S5, supplementary material).

## 4. Discussion

Our results show the effectiveness of the PREVISY in reducing several risk factors for T2D and cardiovascular disease in an indigenous population from northwest Mexico with well-documented health (obesity and high risk for type 2 diabetes development) and social disadvantages, and this has not been reported previously. This achievement was made through the application of translational design, an adaptation of the National Diabetes Prevention Program (NDPP) protocol [29, 30] that was regionalized by taking into account the lifestyle of this particular indigenous group. On this occasion, we were challenged to translate it to the Yaqui indigenous community, the ethnic group most represented in the state of Sonora, Mexico, which has serious obesity problems and a very high risk of chronic diseases, particularly T2D. The PREVISY was evaluated in the short- (6 months) and medium-term periods (12 months), and the percentage of retention was 58.0% and 55.9%, respectively (i.e., the completers). Retention percentages have been reported in interventions of the same nature that ranges from 43.0 to 79.0% [24–26, 34, 43–46], so the retention of the PREVISY is in this range. A lower percentage of male participants was observed (19.4%), which has been reported in other studies [3, 47]. It has been reported that men in this community have long working days, which could explain their low participation [18]. In addition, a lower participation of the indigenous population compared to that of the nonindigenous population in this type of intervention has been reported in other studies [19, 22].

The NDPP program has two main goals: loss of body weight (≥7%) and promotion of physical activity (≥150 minutes/week). In our study, we analyzed three body weight loss goals (5 < 7%, 7 < 10% and ≥10%) since improvements in health have been reported even with a body weight loss of 5% and 3% [48]. According to the ITT, the subjects who achieved the goal of 7% comprised 14.0% of the participants, and according to the PA, the completer subjects that achieved the goal of 7% comprised 24.0% after the intensive phase. The study by Jiang et al. [24] in Native Americans and Alaska Natives reported that 22.5% achieved the 7% goal, similar to the 23.8% reported in the study by Kramer et al. [25]; these percentages are very similar to those found in our study. In the study by Vanderwood et al. [45], the percentage was higher, with 45.0% completer subjects achieving it. It is important to mention that in our study 42.6% of the completer subjects managed to lose ≥5% of their body weight in the intensive phase, improving the health of these participants.

For physical activity, according to the PA, at the end of the intensive phase, 33.3% achieved the goal of 150 minutes/week; however, at some point in this phase, 59.2% met the goal, but they could not maintain it. A similar pattern was observed in the medium term. It is important to mention that subjects did not lose motivation, so they did not stop performing physical activity. In our study, lifestyle trainers gave information about the health benefits of physical activity, and participants made weekly plans for physical activity with the minutes/week to perform and the type of activity they were going to do. In this way, the participants increased their minutes/week of physical activity and the intensity weekly; however, they were not supervised to verify that they met the weekly objectives. In other studies, it has been reported that 66-70% of subjects achieved a short-term goal for physical activity [34, 45]. It should be noted that these studies had scheduled and supervised sessions of physical activity and greater resources with which to promote it among the participants.

The primary outcome established in this study was the change in body weight. According to the ITT, a significant reduction in body weight was found in the short term and this was maintained in the medium term. Compared to studies that promote healthy lifestyles but with more stringent designs, such as randomized and controlled clinical studies, our study demonstrated lower weight loss results [19–22]. However, it has been reported that when translating these programs to the “real world,” body weight loss is less [49]. In a multicenter translational study that applied the DPP protocol [50] and was conducted in five urban clinics in Sonora, Mexico, significant body weight loss (ITT) was reported at 6 months in all clinics (clinic 1: 5.96 kg; clinic 2: 2.11 kg; clinic 3: 1.75 kg; clinic 4: 2.32 kg; and clinic 5: 1.61 kg). In our study, the body weight loss according to the ITT was 2.0 kg at 6 months, which is similar to that reported in some clinics. In part of a review study, it was reported that the mean body weight loss in translational studies using an ITT was 2.8 kg (3.3% of initial body weight) after one year of follow-up [51], which is very similar to that found in our study after one year of follow-up and by the ITT with a mean of -1.9 kg (2.2%) of body weight loss.

According to the PA, the body weight loss was greater at -3.9 kg (4.5%) at 6 and 12 months, so it is comparable with that reported by other studies and in some cases with better results [24–26]. In the study cited above [50], though it used a PA, the body weight loss at 6 months was greater in the five clinics (clinic 1: 7.92 kg; clinic 2: 3.49 kg; clinic 3: 2.76 kg; clinic 4: 5.09 kg; and clinic 5: 3.18 kg) than that using an ITT. These results were similar to the body weight loss of our study, which was -3.9 kg at 6 months of the program using a PA. Furthermore, the authors mention that clinic 1 had a greater body weight loss because a higher percentage of participants achieved the goal of body weight loss than the other clinics, and there could have been greater care in this clinic. This body weight loss has several health benefits, since it has been reported that for one kilogram of body weight loss, the risk of developing T2D is reduced by 16% [52]. Usually, one-third of the body weight loss at 6 months is regained after one year of follow-up [53]. However, our results show that the amount of body weight loss remained, providing participants with better health benefits in the medium term.

Body weight loss reduces the risk of developing T2D and cardiometabolic risk. Some studies have reported a significant body weight loss and other obesity parameters (BMI and waist circumference) but not significant improvements in fasting glucose and lipid profiles; however, biologically, only with body weight loss do these results reduce cardiometabolic risk [54, 55]. Participants in our study reduced their levels of fasting glucose in the short and medium term with an ITT and PA. Several studies have reported decreases in this parameter in the short term; however, fasting glucose tends to rise in the medium term. The improvements in fasting glucose reported in these translation studies range from -4.0 to -7.9 mg/dL [24, 26, 45]. In our study, the results of this variable were better, since the decrease was -7.7 to -13.8 mg/dL according to the ITT and -10.7 to -22.4 mg/dL according to the PA in the short and medium term, respectively, which contributes to the reduction of the risk of T2D.

Regarding lipid profiles, the reported results in our study show significant improvements in the short term. In the medium term, there was a reduction in total cholesterol and triglycerides, but it was not significant. However, the biological maintenance of these improvements in these parameters translates to an improvement in the cardiovascular risk profile of the subjects [24]. Aldana et al. [43], using a PA, reported a significant reduction of total cholesterol in the short term (-17.23 mg/dL), though it was not significant in the medium term (-7.3 mg/dL) and improvements in triglycerides in the short term (-47.64 mg/dL) and in the medium term (-44.27 mg/dL) without significant improvements in HDL-c and LDL-c in either the short or medium term. While in our study, the decrease in total cholesterol and triglycerides was lower than these results, it is important to mention that in this study [43], people undergoing pharmacological treatment for their lipid profiles were not excluded, which could have influenced the greater decrease in the triglyceride levels of total cholesterol in their results than in our results, since in our study, all subjects undergoing pharmacologic treatment for their lipid profiles were excluded. Jiang et al. [24], using a PA, reported significant improvements in triglycerides (-15.5 mg/dL) and LDL-c (-3.9 mg/dL) in the short term and improvements in triglycerides (-6.6 mg/dL), LDL-c (-3.2 mg/dL), and HDL-c (0.9 mg/dL) in the medium term. Compared with those in our study, the improvements in the lipid profiles were smaller but significant due to a larger sample size in the study by Jiang et al. [24] than in our study. Although our lipid profile results were not significant in the medium term, the improvements can be explained biologically, since the cardiovascular risk of the participants was reduced [24].

The obesogenic environment present in the Yaqui community is of the utmost importance, since, from this, several barriers for healthy lifestyle changes arise. Unfortunately, marketing is aggressive and sometimes dominates the diet of this ethnic group, which leads individuals to adopt unfavorable eating habits. By training and standardizing the practices of health coaches, mainly in the diagnosis and treatment of obesity, lifestyle coaches can play an important role in these programs [56]. Currently, lifestyle promotion programs focused on healthy food and the promotion of physical activity are the gold standard for body weight loss and improving cardiometabolic parameters and the consequent reduction of chronic disease risk [57]. It has been shown that changes in lifestyle last a long time, and some studies have reported maintaining favorable results for several years of follow-up [21, 22, 58].

In addition to the improvements in the variables studied, it has also been reported that reductions in body weight in obese subjects show benefits such as improvements in cardiorespiratory condition, blood pressure, systemic inflammation levels, fatty liver, improved quality of life and sexual performance, reduced sleep apnea, urinary incontinence, and symptoms of depression [59].

It is very important to carry out an adequate evaluation to identify the subjects that can benefit most from this type of intervention. Fortunately, our recruitment protocol was extremely careful, successfully recruiting an adequate number of people with overweight/obesity and risk of T2D (by FINRISK), in addition to other specifications already mentioned. Every effort was made to recruit and encourage the adherence of male subjects; however, their participation was a minority, as has been reported in other studies [3, 47]. Some translational research studies mention that it is a limitation not to have a control group; however, it is an application in the “real world,” which should not be a limitation. On the contrary, it should support the implementation of this type of intervention on a large scale, since the results are favorable, and from our point of view, it is unethical to have a control group in this type of study design, where there is strong evidence of favorable results.

It is a great strength of the study that more than ninety percent of the subjects were measured at 6 and 12 months, thus giving greater support to the ITT. To mention some limitations, self-reports of physical activity can overestimate the number of minutes per week the participants performed. The retention percentage was low; however, it is consistent with what has been reported in this type of intervention. The motivation provided by lifestyle coaches is essential to motivate participants. In the case of physical activity, a planned session is recommended for subjects, as the impact on subjects may be greater with supervised physical activity routines some days a week.

## 5. Conclusions

The PREVISY, which focuses on changing lifestyle, is feasible, and its effectiveness is demonstrated in the improvement in improving obesity parameters and cardiovascular risk factors. With this, the intervention reduced the risk of T2D and the cardiometabolic risk in overweight/obese subjects with a risk of T2D. It is a challenge to translate a program of this magnitude to the type of population studied; the Yaqui community is an indigenous ethnic group characterized by socioeconomic barriers that hinder the process. However, the results demonstrate the effectiveness of the PREVISY, so it is feasible to implement the PREVISY with a larger sample size in this indigenous population, and it can be considered as part of a large-scale implementation within the Yaqui community. Future studies should support this type of intervention program because they are the gold standard for the prevention of chronic diseases.

## Figures and Tables

**Table 1:** Sessions of the PREVISY with obesity and diabetes risk.

Intensive phase sessions
(1) Welcome to the program
(2) Be a fat and calorie detective
(3) Three ways to eat less fat and fewer calories
(4) Healthy eating
(5) Move those muscles
(6) Being active, a way of life
(7) Tip the calorie balance
(8) Take charge of what is around you
(9) Problem solving
(10) Four keys to healthy eating out
(11) Talk back to negative thoughts
(12) The slippery of lifestyle change
(13) Jump start your activity plan
(14) Make social cues work for you
(15) You can manage stress
(16) Ways to stay motived
(17) Extra session: menus for holiday parties
Maintenance phase sessions
(1) Welcome to the maintenance phase
(2) Healthy eating with variety and balance
(3) More volume, fewer calories
(4) Stepping up to physical activity
(5) Balance your thoughts for long-term maintenance
(6) Final session: looking back and looking forward

**Table 2:** Sociodemographic, clinical, and anthropometric characteristics of the participants.

Characteristics	Total	Completers	Noncompleters	*p*
*N*	93	54	39	
Gender^‡^				1.00
Female	75 (80.6)	44 (81.5)	31 (79.5)	
Male	18 (19.4)	10 (18.5)	8 (20.5)	
Age (years)^†^	39.5 ± 11.2	40.1 ± 10.5	38.6 ± 12.1	0.54
Read in Spanish (yes)^‡^	86 (92.5)	50 (92.5)	36 (92.3)	1.00
Little	4 (4.3)	2 (3.7)	2 (5.1)	
Write in Spanish (yes)^‡^	83 (89.3)	49 (90.7)	34 (87.1)	0.22
Little	5 (5.4)	1 (1.85)	4 (10.3)	
Scholarship^‡^				0.59
Primary	27 (29.1)	13 (24.1)	14 (35.9)	
Secondary school	36 (38.7)	21 (38.9)	15 (38.5)	
High school	15 (16.1)	10 (18.5)	5 (12.8)	
University	15 (16.1)	10 (18.5)	5 (12.8)	
Civil status^‡^				0.008
Single, widower or separate	31 (33.3)	24 (44.4)	7 (18.0)	
Married or free union	62 (66.7)	30 (55.6)	32 (82.0)	
Overweight^‡^	28 (30.1)	19 (35.2)	9 (23.1)	0.25
Obesity^‡^	65 (69.9)	35 (64.8)	30 (76.9)	0.25
Central obesity^‡^	92 (98.9)	53 (98.2)	39 (100.0)	1.00
Previous diagnosis of HT^‡^	13 (14.0)	8 (14.8)	5 (12.8)	1.00
Family history of diabetes^‡^	85 (91.4)	49 (90.7)	36 (92.3)	0.33
T2D risk (FINDRISC)^‡^				0.51
Moderate risk	33 (35.5)	21 (38.9)	12 (30.8)	
High and very high risk	60 (64.5)	33 (61.1)	27 (69.2)	
Body weight (kg)^†^	85.9 ± 14.6	85.0 ± 13.5	87.1 ± 16.1	0.50
WC (cm)^†^	104.4 ± 10.6	103.3 ± 10.1	106.0 ± 11.3	0.23
BMI (kg/m^2^)^†^	33.2 ± 5.2	32.6 ± 4.6	34.1 ± 6.0	0.17
Body fat (%)^†^	36.1 ± 5.2	36.2 ± 4.7	36.0 ± 5.8	0.90
SBP (mmHg)^†^	115.7 ± 13.0	116.6 ± 11.0	114.5 ± 15.5	0.45
DBP (mmHg)^†^	73.3 ± 9.4	73.6 ± 8.6	72.8 ± 10.5	0.67
Total cholesterol (mg/dL)^†^	169.0 ± 30.8	173.1 ± 33.2	163.3 ± 26.6	0.13
LDL-c (mg/dL)^†^	102.9 ± 25.9	104.9 ± 27.1	100.0 ± 24.2	0.38
HDL-c (mg/dL)^†^	36.8 ± 7.4	37.1 ± 7.7	36.3 ± 7.0	0.59
Triglycerides (mg/dL)^†^	155.7 ± 87.7	159.1 ± 75.2	151.1 ± 103.4	0.66
Fasting glucose (mg/dL)^†^	109.6 ± 26.2	110 ± 28.9	108.7 ± 22.4	0.77
Physical activity (min/wk)^¥^	0 (0, 0)	0 (0, 0)	0 (0, 0)	0.29

WC: waist circumference; SBP: systolic blood pressure; DBP: diastolic blood pressure. ^‡^Expressed as *n* (%). ^†^Expressed as the mean ± SD. ^¥^Expressed as the median and interquartile ranges. *p* value: *t*-test for independent samples (normally distributed variables); Mann–Whitney test (variables with nonnormal distribution); *χ*^2^ test and Fisher's exact tests (categorical variables).

**Table 3:** Comparison of obesity parameters, metabolic markers, and physical activity of the participants (ITT).

Variable	*n*	Basal	Intensive phase	Δ	*n*	Maintenance phase	Δ
Body weight (kg)^†^	93	85.9 ± 14.6	83.8 ± 14.8	−2.0 ± 4.3^∗^	92	84.1 ± 14.6	−1.9 ± 5.1^∗^
WC (cm)^†^	93	104.4 ± 10.6	102.2 ± 11.1	−2.2 ± 5.5^∗^	92	102.8 ± 11.3	−1.7 ± 5.8^∗^
BMI (kg/m^2^)^†^	93	33.3 ± 5.2	32.4 ± 5.3	−0.8 ± 1.7^∗^	92	32.5 ± 5.3	−0.7 ± 2.3^∗^
Total cholesterol (mg/dL)^†^	93	169.0 ± 30.8	160.4 ± 33.3	−8.5 ± 21.0^∗^	92	167.4 ± 32.7	−2.0 ± 21.0
LDL-c (mg/dL)^†^	90	102.9 ± 25.9	93.0 ± 29.4	-9.9 ± 18.1^∗^	89	105.7 ± 28.2	2.3 ± 18.2
HDL-c (mg/dL)^†^	93	36.8 ± 7.4	41.0 ± 8.3	4.2 ± 8.3^∗^	92	34.4 ± 7.8	−2.5 ± 7.0
Triglycerides (mg/dL)^†^	93	155.7 ± 87.7	140.8 ± 68.8	−14.8 ± 64.4^∗^	92	144.1 ± 75.0	−10.9 ± 61.9
Fasting glucose (mg/dL)^†^	93	109.6 ± 26.2	101.8 ± 20.4	−7.7 ± 14.0^∗^	92	100.8 ± 31.5	−8.9 ± 26.7^∗^
Physical activity (min/wk)^¥^	93	0 (0, 0)	0 (0, 90)	0 (0, 90)^∗^	92	0 (0, 30)	0 (0, 30)^∗^

WC: waist circumference. Δ: change value. ^†^Expressed as the mean ± SD. ^¥^Expressed as the median and interquartile ranges. ^∗^*p* ≤ 0.05. Paired *t*-test (normally distributed variables) and Wilcoxon rank-sum test (variables with nonnormal distribution).

**Table 4:** Comparison of obesity parameters, metabolic markers, and physical activity of the participants (PA).

Variable	*n*	Basal	Intensive phase	Δ	*n*	Maintenance phase	Δ
Body weight (kg)^†^	54	85.0 ± 13.5	81.1 ± 12.9	−3.9 ± 4.5^∗^	52	81.1 ± 12.9	−3.9 ± 6.2^∗^
WC (cm)^†^	54	103.3 ± 10.1	99.0 ± 9.5	−4.2 ± 5.8^∗^	52	99.7 ± 9.8	−3.6 ± 6.1^∗^
BMI (kg/m^2^)^†^	54	32.6 ± 4.6	31.0 ± 4.1	−1.5 ± 1.8^∗^	52	31.1 ± 4.2	−1.5 ± 2.4^∗^
Total cholesterol (mg/dL)^†^	54	173.1 ± 33.2	160.4 ± 35.5	−12.6 ± 22.2^∗^	52	172.5 ± 36.3	−1.8 ± 22.9
LDL-c (mg/dL)^†^	53	104.9 ± 27.1	91.2 ± 31.9	−13.6 ± 19.6^∗^	51	108.7 ± 30.6	2.4 ± 18.6
HDL-c (mg/dL)^†^	54	37.1 ± 7.7	43.2 ± 10.6	6.0 ± 9.8^∗^	52	34.9 ± 8.9	2.4 ± 8.0
Triglycerides (mg/dL)^†^	54	159.1 ± 75.2	136.3 ± 66.4	−22.7 ± 63.2^∗^	52	146.6 ± 69.5	−11.8 ± 62.0
Fasting glucose (mg/dL)^†^	54	110.3 ± 28.9	99.5 ± 18.5	−10.7 ± 15.6^∗^	52	96.6 ± 23.4	−14.4 ± 16.0^∗^
Physical activity (min/wk)^¥^	54	0 (0, 0)	55 (0, 160)	47.5 (0, 150)^∗^	52	0 (0, 60)	0 (0, 60)^∗^

WC: waist circumference. Δ: change value. ^†^Expressed as the mean ± SD. ^¥^Expressed as the median and interquartile ranges. ^∗^*p* ≤ 0.05. Paired *t*-test (normally distributed variables) and Wilcoxon rank-sum test (variables with nonnormal distribution).

## Data Availability

The data used to support the findings of this study are available from the corresponding author upon request.

## Supplementary Materials

Supplementary Materials Table S1: no significant differences were found when analyzing the percentage of body fat in the way in which the other variables were analyzed, so we did a subanalysis according to the goal of body weight loss by the participants (Supplementary materials). Tables S2-S5: we also carried out subanalysis of the improvements of the assed parameters, categorizing the completer subjects by overweight and obesity, and other subanalysis categorizing them with moderate risk and high and very high risk of diabetes by the FINSRISC questionnaire. Here, the dose response effect is observed where there are greater benefits in subjects with obesity and higher risk of diabetes.

## References

[1] Sattin R. W., Williams L. B., Dias J. (2016). Community trial of a faith-based lifestyle intervention to prevent diabetes among African-Americans. *Journal of Community Health*.

[2] Organización Mundial de la Salud (OMS) (2018). Obesidad y sobrepeso, datos y cifras. http://www.who.int/es/news-room/fact-sheets/detail/obesity-and-overweight.

[3] Benyshek D. C., Chino M., Dodge-Francis C., Begay T. O., Jin H., Giordano C. (2013). Prevention of type 2 diabetes in urban American Indian/Alaskan native communities: the life in BALANCE pilot study. *Journal of Diabetes Mellitus*.

[4] Saeedi P., Petersohn I., Salpea P. (2019). Global and regional diabetes prevalence estimates for 2019 and projections for 2030 and 2045: results from the International Diabetes Federation Diabetes Atlas, 9^th^ edition. *Diabetes Research and Clinical Practice*.

[5] CDC National Diabetes Statistics Report (2017). Estimates of diabetes and its burden in the United States. https://www.cdc.gov/diabetes/pdfs/data/statistics/national-diabetes-statistics-report.pdf.

[6] Gracey M., King M. (2009). Indigenous health part1: determinants and disease patterns. *Lancet*.

[7] Kirmayer L. L., Brass G. (2016). Adressing global health disparaties among indigenous people. *Lancet*.

[8] Walker J., Lovett R., Kukutai T., Jones C., Henry D. (2017). Indigenous health data and the path to healing. *Lancet*.

[9] Yu C. H. Y., Zinman B. (2007). Type 2 diabetes and impaired glucose tolerance in aboriginal populations: a global perspective. *Diabetes Research and Clinical Practice*.

[10] Escobedo J., Chavira I., Martínez L., Velasco X., Escandón C., Cabral J. (2010). Diabetes and other glucose metabolism abnormalities in Mexican Zapotec and Mixe Indians. *Diabetic Medicine*.

[11] Esparza-Romero J., Valencia M. E., Urquidez-Romero R. (2015). Environmentally driven increases in type 2 diabetes and obesity in Pima Indians and non-Pimas in Mexico over a 15-year period: the Maycoba project. *Diabetes Care*.

[12] Lourenço A. E. P., Santos R. V., Orellana J. D. Y., Coimbra C. E. A. (2008). Nutrition transition in Amazonia: obesity and socioeconomic change in the Suruí Indians from Brazil. *American Journal of Human Biology*.

[13] Herrera-Huerta E. V., García-Montalvo E. A., Méndez-Bolaina E., López-López J. J., Valenzuela O. L. (2014). Sobrepeso y obesidad en indígenas nahuas de Ixtaczoquitlán, Veracruz, México. *Revista Peruana de Medicina Experimental y Salud Pública*.

[14] Cardona-Arias J. A., Llanes-Agudelo O. M. (2013). Hipertensión arterial y sus factores de riesgo en indígenas Emberá-Chamí. *Revista CES Medicina*.

[15] Kolahdooz F., Sadeghirad B., Corriveau A., Sharma S. (2017). Prevalence of overweight and obesity among indigenous populations in Canada: a systematic review and meta-analysis. *Critical Reviews in Food Science and Nutrition*.

[16] Castro-Juarez A. A., Serna-Gutiérrez A., Lozoya-Villegas J. F., Toledo-Dominguez I., Díaz-Zavala R. G., Esparza-Romero J. (2018). Prevalence of previous diagnosis of hypertension and associated factors in the Yaqui indigenous of Sonora. *Revista Mexicana de Cardiología*.

[17] Rodríguez-Morán M., Guerrero-Romero F., Brito-Zurita O. (2008). Cardiovascular risk factors and acculturation in Yaquis and Tepehuanos Indians from Mexico. *Archives of Medical Research*.

[18] Gutiérrez A. S., Romero J. E. (2018). Adaptation and reproducibility of a questionnaire to assess physical activity in epidemiological studies among Yaqui Indians from Sonora, Mexico. *RESPYN Revista de Salud Pública y Nutrición*.

[19] Tuomilehto J., Lindström J., Eriksson J. G. (2001). Prevention of type 2 diabetes mellitus by changes in lifestyle among subjects with impaired glucose tolerance. *The New England Journal of Medicine*.

[20] Pan X. R., Li G. W., Hu Y. H. (1997). Effects of diet and exercise in preventing NIDDM in people with impaired glucose Tolerance: the Da Qing IGT and Diabetes Study. *Diabetes Care*.

[21] Lindström J., Ilanne-Parikka P., Peltonen M. (2006). Sustained reduction in the incidence of type 2 diabetes by lifestyle intervention: follow-up of the Finnish Diabetes Prevention Study. *Lancet*.

[22] Knowler W. C., Barrett-Connor E., Fowler S. E. (2002). Reduction in the incidence of type 2 diabetes with lifestyle intervention or metformin. *The New England Journal of Medicine*.

[23] Albright A. L., Gregg E. W. (2013). Preventing type 2 diabetes in communities across the U.S. *American Journal of Preventive Medicine*.

[24] Jiang L., Manson S. M., Beals J. (2013). Translating the diabetes prevention program into American Indian and Alaska Native communities: results from the special diabetes program for Indians Diabetes Prevention demonstration project. *Diabetes Care*.

[25] Kramer M. K., Kriska A. M., Venditti E. M. (2009). Translating the diabetes prevention program. *American Journal of Preventive Medicine*.

[26] Boltri J. M., Davis-Smith Y. M., Seale J. P., Shellenberger S., Okosun I. S., Cornelius M. E. (2008). Diabetes prevention in a faith-based setting. *Journal of Public Health Management and Practice*.

[27] Urquidez-Romero R., Esparza-Romero J., Chaudhari L. S. (2014). Study design of the maycoba project: obesity and diabetes in mexican pimas. *American Journal of Health Behavior*.

[28] Lindström J., Tuomilehto J. (2003). The diabetes risk score: a practical tool to predict type 2 diabetes risk. *Diabetes Care*.

[29] CDC Centers of Disease Control and Prevention (2015). National Diabetes Prevention Program. Standard and Operating Procedures. http://www.cdc.gov/diabetes/prevention/pdf/dprp-standards.pdf.

[30] CDC Centers of Disease Control and Prevention (2017). National Diabetes Prevention Program. https://www.cdc.gov/diabetes/spanish/prevention/index.html.

[31] Gutiérrez A. S., Esparza-Romero J. (2019). Diseño y validación de un cuestionario de frecuencia de consumo de alimentos para evaluar la dieta en indígenas Yaquis de Sonora, México. *Acta Universitaria*.

[32] CDC Centers of Disease Control and Prevention (2012). National Diabetes Prevention Program. 2012-A CDC-Developed Curriculum and Handouts (Spanish). https://www.cdc.gov/diabetes/prevention/resources/curriculum.html.

[33] CDC Centers of Disease Control and Prevention (2012). National Diabetes Prevention Program. 2012-B CDC-Developed Curriculum and Handouts (Spanish). https://www.cdc.gov/diabetes/prevention/resources/curriculum.html.

[34] Amundson H. A., Butcher M. K., Gohdes D. (2009). Translating the diabetes prevention program into practice in the general community. *The Diabetes Educator*.

[35] Aguilar A. C. G., Romero J. E., Martínez H. H. H. (2018). Agreement between HemoCue and glucose oxidase methods for blood glucose measurement in a field work study of diabetes: the Comcaac project. *Biotecnia*.

[36] WHO World Health Organization (2018). Body mass index- BMI. http://www.euro.who.int/en/health-topics/disease-prevention/nutrition/a-healthy-lifestyle/body-mass-index-bmi.

[37] Lukaski H. C., Johnson P. E., Bolonchuk W. W., Lykken G. I. (1985). Assessment of fat-free mass using bioelectrical impedance measurements of the human body. *The American Journal of Clinical Nutrition*.

[38] Rising R., Swinburn B., Larson K., Ravussin E. (1991). Body composition in Pima Indians: validation of bioelectrical resistance. *The American Journal of Clinical Nutrition*.

[39] Pickering T. G., Hall J. E., Appel L. J. (2005). Recommendations for blood pressure measurement in humans and experimental animals: part 1: blood pressure measurement in humans: a statement for professionals from the subcommittee of professional and public education of the American Heart Association Council on high blood pressure research. *Circulation*.

[40] ENSANUT MC Encuesta Nacional de Salud y Nutrición de Medio Camino (2016). *Primera Edición*.

[41] Ainsworth B. E., Haskell W. L., Herrmann S. D. (2011). 2011 compendium of physical activities. *Medicine and Science in Sports and Exercise*.

[42] Friedewald W. T., Levy R. I., Fredrickson D. S. (1972). Estimation of the concentration of low-density lipoprotein cholesterol in plasma, without use of the preparative ultracentrifuge. *Clinical Chemistry*.

[43] Aldana S., Barlow M., Smith R. (2016). A worksite diabetes prevention program. *AAOHN Journal*.

[44] Katula J. A., Vitolins M. Z., Rosenberger E. L. (2011). One-year results of a community-based translation of the diabetes prevention program: Healthy-Living Partnerships to Prevent Diabetes (HELP PD) project. *Diabetes Care*.

[45] Vanderwood K. K., Hall T. O., Harwell T. S., Butcher M. K., Helgerson S. D., on behalf of the Montana Cardiovascular Disease and Diabetes Prevention Program Workgroup (2010). Implementing a state-based cardiovascular disease and diabetes prevention program. *Diabetes Care*.

[46] Davis-Smith Y. M., Boltri J. M., Seale J. P., Shellenberger S., Blalock T., Tobin B. (2007). Implementing a diabetes prevention program in a rural African-American church. *Journal of the National Medical Association*.

[47] Absetz P., Valve R., Oldenburg B. (2007). Type 2 diabetes prevention in the “real world”. One-year results of the GOAL implementation trial. *Diabetes Care*.

[48] Mau M. K., Keawe'aimoku Kaholokula J., West M. R. (2010). Translating diabetes prevention into Native Hawaiian and Pacific islander communities: the PILI’Ohana Pilot Project. *Progress in Community Health Partnerships*.

[49] Saaristo T., Moilanen L., Korpi-Hyovalti E. (2010). Lifestyle intervention for prevention of type 2 diabetes in primary health care: one-year follow-up of the Finnish National Diabetes Prevention Program (FIN-D2D). *Diabetes Care*.

[50] Armenta-Guirado B. I., Martínez-Contreras T., Candia-Plata M. C. (2019). Effectiveness of the Diabetes Prevention Program for obesity treatment in real world clinical practice in a middle-income country in Latin America. *Nutrients*.

[51] Norris S. L., Zhang X., Avenell A. (2005). Long-term effectiveness of weight-loss interventions in adults with pre-diabetes; a review. *American Journal of Preventive Medicine*.

[52] Dunkley A. J., Bodicoat D. H., Greaves C. J. (2014). Diabetes prevention in the real world: effectiveness of pragmatic lifestyle interventions for the prevention of type 2 diabetes and of the impact of adherence to guideline Recommendations. *Diabetes Care*.

[53] Wing R. R., Tate D. F., Gorin A. A., Raynor H. A., Fava J. L. (2006). A self-regulation program for maintenance of weight loss. *The New England Journal of Medicine*.

[54] Ackermann R. T., Finch E. A., Brizendine E., Zhou H., Marrero D. G. (2008). Translating the Diabetes Prevention Program into the community: the DEPLOY Pilot Study. *American Journal of Preventive Medicine*.

[55] McTigue K. M., Conroy M. B., Bigi L., Murphy C., McNeil M. (2009). Weight loss through living well. *The Diabetes Educator*.

[56] Díaz-Zavala R. G., Armenta-Guirado B. I., Martínez-Contreras T. D. J. (2017). Translational study of obesity management using the Diabetes Prevention Program “Group Lifestyle Balance” in primary care clinics and public hospitals from Mexico: study protocol. *Revista Española de Nutrición Humana y Dietética*.

[57] Jensen M. D., Ryan D. H., Apovian C. M. (2014). 2013 AHA/ACC/TOS guideline for the management of overweight and obesity in adults. *Circulation*.

[58] Li G., Zhang P., Wang J. (2008). The long-term effect of lifestyle interventions to prevent diabetes in the China Da Qing Diabetes Prevention Study: a 20-year follow-up study. *Lancet*.

[59] Pi-Sunyer X. (2014). The look AHEAD trial: a review and discussion of its outcomes. *Current Nutrition Reports*.

