# Supplementary material for: Effectiveness of the Healthy Lifestyle Promotion Program for Yaquis with Obesity and Risk of Diabetes in the Short and Medium Term: A Translational Study
Source: J Diabetes Res. 2020 Sep 29;2020:6320402. doi: 10.1155/2020/6320402 (PMC7545430; doi:10.1155/2020/6320402)
Supplement: Supplementary Materials — Table S1: no significant differences were found when analyzing the percentage of body fat in the way in which the other variables were analyzed, so we did a subanalysis according to the goal of body weight loss by the participants (Supplementary materials). Tables S2-S5: we also carried out subanalysis of the improvements of the assed parameters, categorizing the completer subjects by overweight and obesity, and other subanalysis categorizing them with moderate risk and high and very high risk of diabetes by the FINSRISC questionnaire. Here, the dose response effect is observed where there are greater benefits in subjects with obesity and higher risk of diabetes. [file 6320402.f1.pdf]

Supplementary Materials

Table S1: Comparison of percentage of body fat according to the achieved goals of body weight loss.

	Percentage of body fat (%)							
**Goal of body weight loss (%)**	n	Basal	Intensive phase	Δ	n	Basal	Maintenance phase	Δ
**5 < 7**	11	35.6 ± 5.4	35.0 ± 6.0	-0.5 ± 1.3	11	37.7 ± 5.4	36.7 ± 5.7	-1.0 ± 1.0*
**7 < 10**	6	40.2 ± 1.6	39.9 ± 1.4	-0.2 ± 1.2	4	35.8 ± 2.6	34.3 ± 4.0	-1.4 ± 1.8
**≥ 10**	7	38.1 ± 2.5	35.4 ± 3.7	-2.6 ± 1.5*	10	39.6 ± 2.1	36.1 ± 3.5	-3.5 ± 2.0*

Δ: Change value; Expressed as mean ± SD; *: p≤0.05. Paired t-test (normally distributed variables).

Table S2: Comparison of obesity parameters and physical activity in completers participants with overweight and obesity.

Variables	Overweight (n=19)	Obesity (n=35)	Δ Overweight	Δ Obesity
**Body weight (kg) †**				
Basal	74.3 ± 8.3	90.8 ± 12.3		
Intensive phase	72.3 ± 8.2	85.8 ± 12.5	-2.0 ± 1.9*	-5.0 ± 5.2*
Maintenance phase	72.8 ± 9.2	85.5 ± 12.6	-1.3 ± 1.9*	-5.3 ± 7.2*
**WC (cm) †**				
Basal	94.8 ± 6.4	107.9 ± 8.7		
Intensive phase	92.5 ± 5.2	102.5 ± 9.4	-2.2 ± 4.1*	-5.6 ± 6.4*
Maintenance phase	93.8 ± 6.4	102.9 ±9.8	-1.0 ± 3.1	-5.0 ± 6.8*
**BMI (kg/m^2) †**				
Basal	28.3 ± 1.1	34.9 ± 4.1		
Intensive phase	27.6 ± 1.2	32.9 ± 4.0	-0.7 ± 0.7*	-1.9 ± 2.0*
Maintenance phase	27.8 ± 0.7	32.8 ± 4.2	-0.5 ± 0.7*	-2.0 ± 2.8*
**Physical activity (min/wk) ¥**				
Basal	0 (0, 0)	0 (0, 0)		
Intensive phase	45 (0, 150)	60 (0, 220)	45 (0, 120)*	60 (0, 195)*
Maintenance phase	30 (0, 150)	0 (0, 15)	50 (0, 100)*	0 (0, 0)*

WC: Waist circumference. Δ: Change value; †: Expressed as mean ± SD; ¥: Expressed as median and interquartile ranges. *: p≤ 0.05. Paired t-test (normally distributed variables) and Wilcoxon rank-sum test (variables with non-normal distribution).

Table S3: Comparison of metabolic markers in completers participants with overweight and obesity.

Variables	Overweight (n=19)	Obesity (n=35)	Δ Overweight	Δ Obesity
**Total cholesterol (mg/dL)**				
Basal	175.5 ± 35.8	171.8 ± 32.1		
Intensive phase	170.3 ± 41.4	155.0 ± 31.2	-5.2 ± 18.4	-16.7 ± 23.3*
Maintenance phase	174.7 ± 35.7	171.3 ± 37.1	-3.5 ± 13.5	-1.0 ± 26.7
**LDL-c (mg/dL)**				
Basal	107.3 ± 28.7	103.7 ± 26.6		
Intensive phase	107.1 ± 31.7	83.1 ± 29.1	-0.1 ± 15.4	-20.6 ± 18.0*
Maintenance phase	109.4 ± 33.6	108.3 ± 29.5	-0.5 ± 16.3	4.0 ± 19.6
**HDL-c (mg/dL)**				
Basal	37.0 ± 9.5	37.2 ± 6.7		
Intensive phase	38.4 ± 9.7	45.7 ± 10.3	1.4 ± 9.2	8.5 ± 9.3*
Maintenance phase	32.9 ± 7.3	36.0 ± 9.6	-4.8 ± 7.4	-1.1 ± 8.1
**Triglycerides (mg/dL)**				
Basal	171.1 ± 89.4	152.6 ± 67.1		
Intensive phase	148.5 ± 65.5	129.7 ± 66.8	-22.5 ± 55.6	-22.8 ± 67.7*
Maintenance phase	171.3 ± 66.1	133.5 ± 68.7	2.5 ± 67.4	-19.4 ± 58.5
**Fasting glucose (mg/dL)**				
Basal	111.9 ± 43.0	109.4 ± 17.9		
Intensive phase	102.1 ± 27.5	98.0 ± 11.3	-9.7 ± 18.9*	-11.3 ± 13.7*
Maintenance phase	103.1 ± 35.1	93.2 ± 13.3	-9.8 ± 15.6*	-16.7 ± 15.9*

Δ: Change value; Expressed as mean ± SD; *: p≤ 0.05. Paired t-test (normally distributed variables).

Table S4: Comparison of obesity parameters and physical activity in completers participants with moderate, high and very high risk of diabetes.

Variables	Moderate risk (n=21)	High and very high risk (n=33)	Δ Moderate risk	Δ High and very high risk
**Body weight (kg) †**				
Basal	80.3 ± 12.0	88.0 ± 13.8		
Intensive phase	78.0 ± 11.7	83.0 ± 13.3	-2.3 ± 2.3*	-4.9 ± 5.3*
Maintenance phase	78.8 ± 11.8	82.5 ± 13.6	-1.6 ± 3.3*	-5.3 ± 7.2*
**WC (cm) †**				
Basal	98.3 ± 8.8	106.5 ± 9.8		
Intensive phase	96.2 ± 7.9	100.8 ± 10.1	-2.0 ± 4.7*	-5.6 ± 6.1*
Maintenance phase	97.2 ± 8.0	101.3 ± 10.5	-1.3 ± 4.0	-5.1 ± 6.7*
**BMI (kg/m^2) †**				
Basal	30.2 ± 3.4	34.0 ± 4.6		
Intensive phase	29.3 ± 3.2	32.1 ± 4.3	-0.9 ± 0.9*	-1.9 ± 2.0*
Maintenance phase	29.7 ± 3.5	32.0 ± 4.5	-0.6 ± 1.2*	-2.1 ± 2.8*
**Physical activity (min/wk) ¥**				
Basal	0 (0, 0)	0 (0, 0)		
Intensive phase	0 (0, 150)	80 (0, 220)	0 (0, 60)*	80 (0, 195)*
Maintenance phase	0 (0, 135)	0 (0, 30)	0 (0, 110)*	0 (0, 30)*

WC: Waist circumference. Δ: Change value; †: Expressed as mean ± SD; ¥: Expressed as median and interquartile ranges. *: p≤ 0.05. Paired t-test (normally distributed variables) and Wilcoxon rank-sum test (variables with non-normal distribution).

Table S5: Comparison of metabolic markers in completers participants with moderate, high and very high risk of diabetes.

Variables	Moderate risk (n=21)	High and very high risk (n=33)	Δ Moderate risk	Δ High and very high risk
**Total cholesterol (mg/dL)**				
Basal	174.5 ± 40.0	172.1 ± 28.7		
Intensive phase	167.4 ± 26.6	156.0 ± 33.5	-7.1 ± 26.6	-16.1 ± 18.5*
Maintenance phase	177.0 ± 40.1	169.0 ± 34.0	0.1 ± 23.5	-3.0 ± 22.7
**LDL-c (mg/dL)**				
Basal	105.6 ± 30.9	104.5 ± 25.0		
Intensive phase	97.9 ± 28.1	87.3 ± 33.7	-7.7 ± 19.3	-17.2 ± 19.2*
Maintenance phase	110.6 ± 33.4	107.5 ± 29.4	2.6 ± 21.3	2.3 ± 17.0
**HDL-c (mg/dL)**				
Basal	37.4 ± 8.0	37.0 ± 7.6		
Intensive phase	41.8 ± 11.6	44.0 ± 10.2	4.4 ± 9.1*	7.0 ± 10.2*
Maintenance phase	35.7 ± 6.8	34.5 ± 10.1	-2.4 ± 4.9	-2.4 ± 9.5
**Triglycerides (mg/dL)**				
Basal	170.5 ± 99.6	151.8 ± 55.1		
Intensive phase	158.0 ± 84.6	122.5 ± 48.1	-12.4 ± 82.9	-29.3 ± 46.9*
Maintenance phase	161.9 ± 93.0	137.1 ± 49.1	-6.5 ± 57.0	-15.1 ± 65.5
**Fasting glucose (mg/dL)**				
Basal	110.8 ± 40.1	110.0 ± 19.5		
Intensive phase	103.5 ± 24.5	96.9 ± 13.2	-7.2 ± 19.2	-13.0 ± 12.5*
Maintenance phase	101.4 ± 32.3	93.7 ± 15.4	-10.3 ± 14.8*	-16.9 ± 16.4*

Δ: Change value; Expressed as mean ± SD; *: $p \leq 0.05$. Paired t-test (normally distributed variables).
